# Back to BaySICS: A User-Friendly Program for Bayesian Statistical Inference from Coalescent Simulations

**DOI:** 10.1371/journal.pone.0098011

**Published:** 2014-05-27

**Authors:** Edson Sandoval-Castellanos, Eleftheria Palkopoulou, Love Dalén

**Affiliations:** 1 Department of Bioinformatics and Genetics, Swedish Museum of Natural History, Stockholm, Sweden; 2 Department of Zoology, Stockholm University, Stockholm, Sweden; University of Florence, Italy

## Abstract

Inference of population demographic history has vastly improved in recent years due to a number of technological and theoretical advances including the use of ancient DNA. Approximate Bayesian computation (ABC) stands among the most promising methods due to its simple theoretical fundament and exceptional flexibility. However, limited availability of user-friendly programs that perform ABC analysis renders it difficult to implement, and hence programming skills are frequently required. In addition, there is limited availability of programs able to deal with heterochronous data. Here we present the software BaySICS: Bayesian Statistical Inference of Coalescent Simulations. BaySICS provides an integrated and user-friendly platform that performs ABC analyses by means of coalescent simulations from DNA sequence data. It estimates historical demographic population parameters and performs hypothesis testing by means of Bayes factors obtained from model comparisons. Although providing specific features that improve inference from datasets with heterochronous data, BaySICS also has several capabilities making it a suitable tool for analysing contemporary genetic datasets. Those capabilities include joint analysis of independent tables, a graphical interface and the implementation of Markov-chain Monte Carlo without likelihoods.

## Introduction

The power of population genetics and genomics to infer past evolutionary processes has vastly increased in the last 30 years due to the synergy created by highly influential advances, both theoretical (coalescent theory, Bayesian statistics) and technological (high throughput sequencing, high performance computers, ancient DNA analysis) [1], [2], [3].

While coalescent theory has created a simple, powerful, and elegant way to model evolutionary processes [1], [4], Bayesian statistics have provided a solid theoretical framework for the treatment of complex systems as well as for inference based on computer simulations [5], [6], [7]. Furthermore, sequencing of large genomic regions has significantly enhanced statistical power due to larger nucleotide sampling [3], [8], [9]. In addition, the recent progress made in the field of ancient DNA has provided the opportunity to directly trace microevolutionary changes in macrobiotic systems [10], [11].

The most relevant advances in statistical inference have been brought about by the diversification and refinement of Monte Carlo methods, usually exploited by Bayesian methodologies. The most successful technique used for Bayesian inference is the Markov chain Monte Carlo (MCMC) [6]. Despite their success, MCMC methods suffer from known limitations when systems become highly complex, since the calculation of likelihoods, which is indispensable for its implementation, becomes difficult or impossible [12], [13], (but see [14]).

Approximate Bayesian computation (ABC) stands among the most promising Monte Carlo techniques [2], [6], [12], and is increasing in popularity due to its simple fundament and exceptional flexibility, enabled by its likelihood-free implementation [13]. Applications of ABC range from assessing models in human evolution [15], [16], estimating the rate of spread in pathogens [17], [18], to estimation of mutation rates [19], migration rates [20], selection rates [21], and population admixture [22]. In the case of studies that include ancient DNA data, ABC has been successfully used to link environmental events with population structuring [23], [24], past migration events [25], and extinction [26]. Moreover, ABC has been suggested for applications beyond the field of population genetics [12], [13].

Despite its recognized potential, ABC also accounts for well identified challenges, including the necessity for validation and adjustment [2], [12], [13], [27], the choice of summary statistics that are Bayes sufficient (i.e. that fully capture the information contained in the data) [12], [28], and the unreliability in model choice [28], [29]. However, perhaps the most important downside of the ABC method is its low computational efficiency [30].

Many efforts have been made to provide tools that improve the computational efficiency of the analysis [2], [14], [30], [31], [32], [33], as well as to establish a user-friendly interface for wider use [27], [34], [35], [36], [37]. However, the number of options is still limited for evolutionary studies employing heterochronous data. For instance, at present, there is only one program available that provides a single platform for performing simulations, rejection procedures and posterior probabilities estimations for heterochronous data (DIYABC; [38]).

Here we present the software BaySICS: Bayesian Statistical Inference of Coalescent Simulations. A Windows program that provides an integrated and user-friendly platform to perform coalescent simulations for DNA sequence data and ABC analysis including estimation of posterior densities for population parameters and Bayes factors for model comparisons. BaySICS implements a number of tools for improving the simulations and interpretation of results, including novel summary statistics specific for ancient DNA data, 2-D and 3-D graphics, as well as an MCMC-without-likelihoods algorithm.

## Materials and Methods

### The software

BaySICS comprises a set of three independent programs that are managed by a common graphical interface. The first program executes coalescent simulations, the second program performs the post-simulation analysis, and the third program performs validation procedures based on pseudo-observed data sets (PODs).

The first program generates simulated samples corresponding in number, ages and population assignment (if applicable) to the observed ones, under a population history that is fully specified in an input file. It can simulate non-recombining DNA from a locus that is autosomal haploid, diploid or haplo-diploid (X-linked), as well as an Y-linked or mitochondrial. The simulation design consists of an assembly of coalescing blocks, rather than events, delimited by specific time bounds. Each block coalesces lineages from one or two sources (populations or blocks), which permits the simulation of any historical population structure, including populations that merge and split in time. Each block contains its own effective population size (*N_e_*) and growth rate. Ages of the samples, *N_e_*, growth rate, age of a block (equivalent to the age of an event), migration rates, proportion of lineages sourced to a block, generation time, mutation rates, gamma parameter (shape), and transition/transversion bias, are parameters that can be drawn from prior densities. The summary statistics estimated for each simulated data set include the number of total and private haplotypes, gene and nucleotide diversities, *F_ST_*, average number of pairwise differences (within and between populations) and Tajima's *D*. Moreover, multi-dimensional summary statistics can be calculated and employed, such as mismatch distributions, Neighbour-Joining trees and the matrix of pairwise differences among sequences. For heterochronous genetic data, we have further included a novel temporally weighted matrix of pairwise differences. In this matrix, the temporal distances among individuals determine the weight of their pairwise differences, with large temporal distances contributing less to the inference. This summary statistic is useful for tracking demographic changes through time when heterochronous samples with a large temporal coverage are included. The rationale behind this approach is that in studies with wide temporal coverage, the tracking of demographic change through time is improved by discarding information that becomes meaningless due to an excessive temporal separation among samples.

The post-simulation analysis program performs two types of analyses: parameter estimation and model comparison. Both can be performed employing different tables obtained independently and integrated into a single analysis, which provides flexibility for parallelizing the simulations.

The parameter estimation algorithm follows the one proposed by Beaumont et al. [2]. In BaySICS it consists of: *i*) reading the summary statistics and parameters from one or more reference tables; *ii*) applying the rejection procedure (see next paragraph); *iii*) adjusting and estimating the posterior densities of the parameters with optional regression and kernel procedures as described in [2]; *iv*) displaying and saving the posteriors of the parameters of interest (including joint posteriors if required); and *v*) displaying optional charts of the predictive distributions of the summary statistics.

Most of the available software for ABC analyses performs model comparisons by choosing a target number of simulations that have the shortest Euclidean distances to the observed summary statistics. This method, from here on called the proportion method, bypasses the problematic necessity to define an appropriate threshold for rejection. However, when too many summary statistics are employed, a high dimensionality phenomenon can occur, in which a small Euclidean distance (calculated over all the summary statistics) could be accompanied by not-so-small individual distances in some of the summary statistics. In other words, if many summary statistics are employed it is possible that the accepted simulations give values that could be considered unacceptable for some summary statistics although their overall Euclidean distance is still among the shortest ones. If some summary statistics should be kept under an acceptable range, a definition of acceptance thresholds for all the summary statistics could be preferable over the proportion method. BaySICS can perform both methods and, for model choice analysis, repeats the analysis 50 times for different proportions or thresholds in order to assess the consistency of the acceptance ratios at different tolerance levels. The ratios of the different models in the accepted sample is then interpreted as an estimation of the models likelihoods from which it is straightforward to estimate Bayes factors. The procedure can also be improved by applying a logistic regression, also implemented in BaySICS, where the summary statistics are seen as explanatory variables of the model likelihoods in a neighbourhood of the observed values [15].

**Figure 1:**
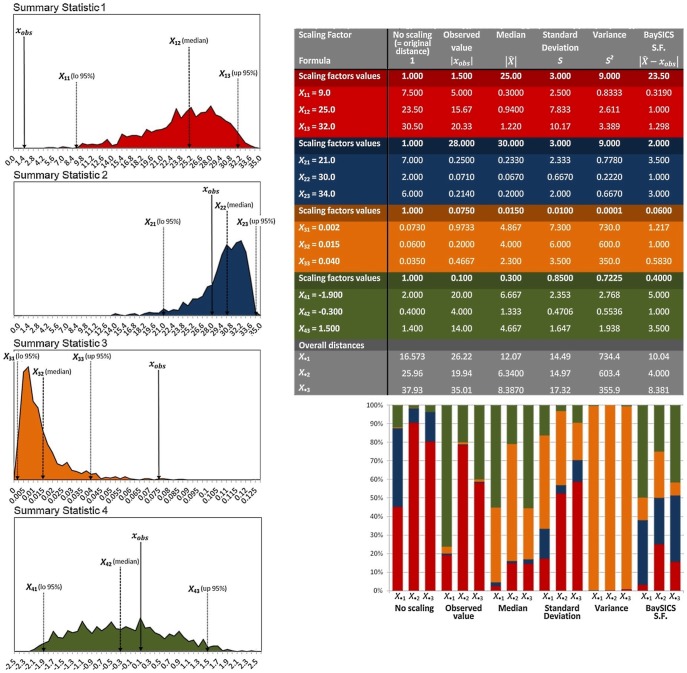
Comparison of different standardization factors for the summary statistics. The charts at left show the probability distribution of four hypothetical summary statistics (before applying any rejection or adjustment) that were chosen to show the properties of six normalizing factors corresponding to the columns in the table at the right. For each summary statistic, three values (*x*_1_, *x*_2_, and *x*_3_) were used to calculate the distances between them and the observed value and then to adjust those distances by the different normalizing factors. The three points, *x*_1_, *x*_2_, and *x*_3_, were chosen to coincide with the quantiles of 2.5%, 50% (the median) and 97.5% respectively of the distributions of the summary statistics, in order to induce the behaviour of the gross of the simulated values. The table shows the values of *x*_1_, *x*_2_, and *x*_3_ when standardized by the different normalizing factors and the bars below illustrate the proportional contribution of each summary statistic (identified by colour) to the overall composite Euclidean distance that would result if those summary statistics corresponded to the ones used for performing an hypothetical ABC analysis.

The calculation of distances between the summary statistics of observed and simulated data is an essential step in the post-simulation analysis. The standard distance is the Euclidean distance:


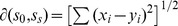
, 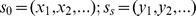, where *s_o_* and *s_s_* are the vectors of summary statistics in the observed and simulated alignments respectively. This distance is usually normalized by the standard deviation of the simulated values [2], [38], in order to counteract the effect of the very different scales that summary statistics can have; for instance the number of segregating sites could be seven orders of magnitude larger than nucleotide diversity. BaySICS employs the difference between the median of the simulated data and the observed value as the normalizing factor, instead of the standard deviation, because this promotes a balanced contribution of every summary statistic in circumstances where other normalizing factors fail to do so. For instance, the examples used in figure 1, show that using the observed value as a standardizing factor produces an overrepresentation of summary statistics 1 and 4 (red and green) in the Euclidean distance because the observed value was small compared to most of the simulated values. Standard deviation also has the disadvantage of neglecting the observed value, such that a large difference between the observed value and the simulated ones (in comparison to the standard deviation) will make the distances large (see summary statistic 1 in red; figure 1). The standardizing factor implemented in BaySICS has the advantage of normalizing to equal values the distances of the most frequent simulated values: the ones around the median.

Pseudo Observed Datasets (PODs) have been proposed as a mean to assess the performance of the ABC methods [12]. By considering a random simulated dataset as the “real” data, for which associated parameters or models are the “real” ones, and performing parameter inference or model choice using the remaining simulated datasets, it is possible to obtain a plethora of measures for the performance of the analysis [13], [38]. Most programs recycle the simulations of the reference table (the one used for inference) as PODs, as a way to save time. In BaySICS, the pseudo-observed data sets are simulated independently for two reasons: 1) only 100–1000 PODs are routinely used, which would imply a trivial investment of computational time; and 2) this widens the utility of PODs for the estimation of, not only statistical power or error, but also statistical robustness, if the PODs are simulated with relaxed assumptions that were not so in the main simulations. In our experience, the impact of the assumptions on the results is a primary source of concern in the application of statistical inference to evolutionary studies. A PODs analysis can be employed in BaySICS for estimating the rate of false positives or false negatives in a model choice analysis, which could be interpreted as statistical power or error type I-II, as well as to obtain measures of the performance in a parameter estimation analysis. Performance measures include the mean relative bias, the square root of the relative mean square error (RRMSE), the 50% and 95% coverage, and the factor 2 [38].

BaySICS also implements an MCMC-without-likelihoods procedure (MCMCwL). This procedure follows Marjoram et. al [14] and performs a pre-run of 10,000 simulations to estimate a threshold that accepts 1% of the simulations and then use that threshold to reject on-the-go the simulations with probability equivalent to the ratio of the transition kernel evaluated in the current state and the newly proposed one [14]. The transition kernel is settled to a normal density with a S.D. being one tenth of the range of the parameter in the prior. The reference table that is obtained by this type of analysis lists the chosen summary statistics and the simulations that were accepted on-the-go with an acceptance threshold that would retain approximately 1% of the most similar simulations (to the observed data) in a normal run.

### Validation and Tests

To assess the features and performance of BaySICS, we carried out an evaluation at three levels: 1) a qualitative assessment; 2) a comparison with theoretical predictions; and 3) an estimation of measures of performance by means of PODs.

The qualitative assessment consisted of a subjective comparison of the features of the software after performing complete analyses on simulated data that were inspired by real case studies. The complete analyses included setting up and running the coalescent simulations to performing parameter estimation and model comparisons. For parameters estimation a relatively complex model was employed including a population structure of three populations, two splits events and nine unknown parameters while the model comparison assessed four hypotheses of demographic history in four time periods (see figure S1).

Theoretical predictions were assessed by using a group of test examples for which the expectations of the times to the first, second, etc. coalescences; the expectation of the time to the most recent common ancestor (*T_MRCA_*), and the expectation of the tree length were calculated according to the formulas in [39]. The theoretical expectations were compared with the average values obtained from 10 000 simulations ran under the same scenario and parameter values with two software options: BaySICS and Bayesian Serial Simcoal (BSSC) [40]. In the case of BSSC only *T_MRCA_* was obtained. The employed test examples included simple scenarios where formulas could be applied in a more or less straightforward way (see table S1). In addition, the estimated summary statistics were compared to the ones obtained from other software (Arlequin [41]). Posterior and predictive distributions obtained by MCMCwL were also compared with the ones obtained by an ordinary analysis without MCMCwL.

**Figure 2:**
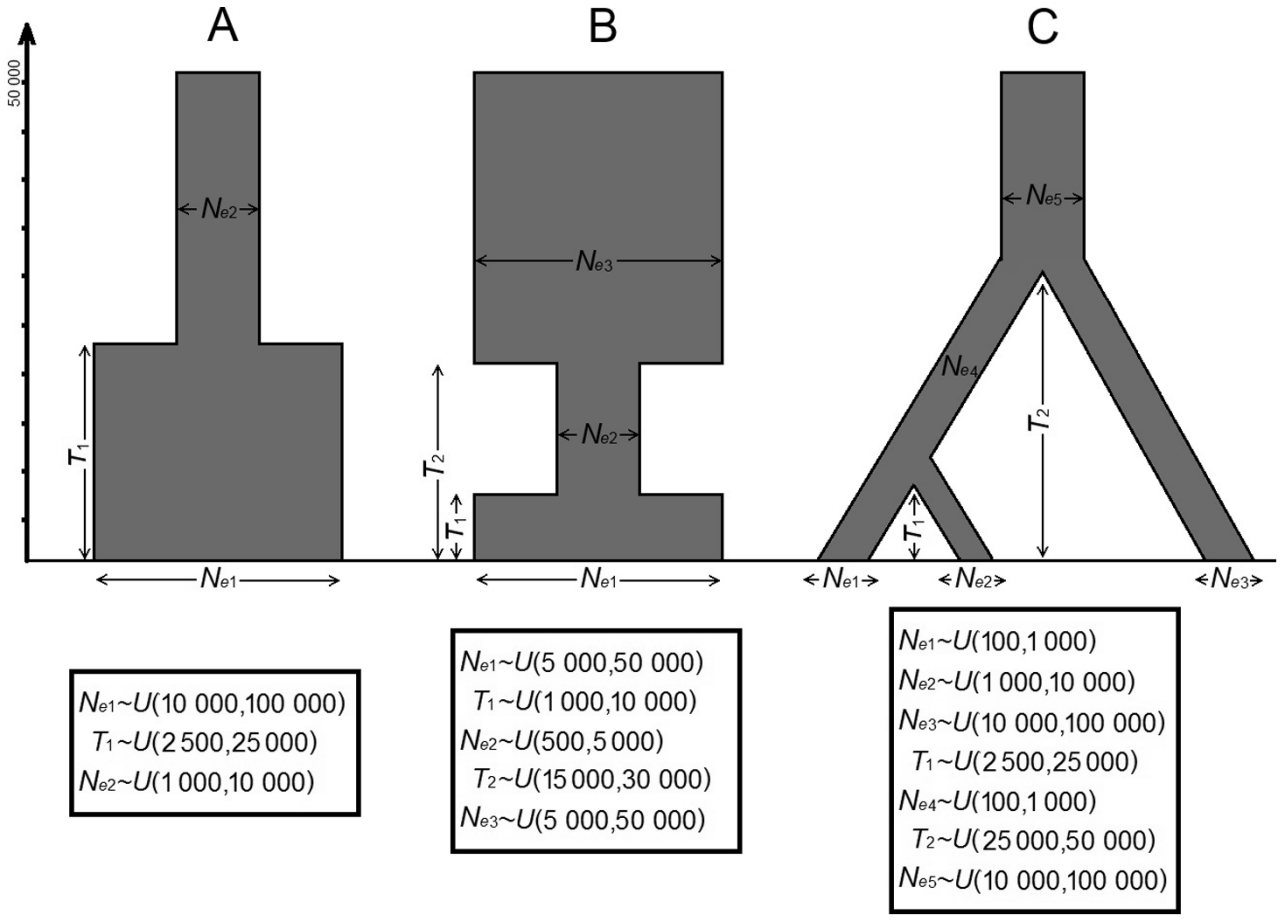
Simulated examples employed for the performance assessment analysis. They were termed simulated example 1(A), simulated example 2 (B) and simulated example 3(C) in the text. A) The first scenario consists of a single change in population size; B) the second scenario consists of a bottleneck followed by population recovery; and C) the third scenario represents a population structure with three populations. The sampling consisted of 51 contemporary haploid individuals in (A); three samples of 17 haploid individuals each, which were taken at present, 15 000 and 35 000 generations before present, in (B); and three samples of 17 haploid individuals each, taken at present, 2 500 and 5 000 generations before present, in (C). The mutation rate was set to 0.15/nucleotide site/10^6^y, the transition/transversion bias was 0.875 and the gamma shape parameter was 0.15. The DNA sequences were l 000 bp long. Boxes show the parameters and prior densities which they were sampled from (*U*(*a*,*b*) means uniform distribution in the interval *a* to *b*).

The performance measurements were obtained by running sets of 10 000 PODs. PODs were obtained by simulating datasets with different combinations of parameter values to assess at once the expected performance of the estimations for a range of parameter values instead of a single combination of values. Each one of those 10 000 PODs were employed to use their parameters as the real values and their summary statistics as the observed values for which a parameter estimation by ABC was performed. Then, the mean relative bias, the square root of the mean square error (RRMSE), the 50% and 95% coverage, and the factor 2 were calculated upon the 10 000 iterations (PODs). Notice that the interpretation of some performance measures, such as RRMSE is slightly different when obtained from PODs with different (real) parameter values, than if the 10 000 PODs were obtained with the same parameter values; however, the interpretation is still useful as the procedure can be seen as an integration over the parametric space of the simulated parameter values and the obtained performance measures are thus the statistical expectation of those measures. In this way, we can assess the performance of the parameters estimation over a parametric space, instead of a single combination of values, for a more general interpretation. Two groups of performance measures were obtained, one using the mode of the posterior distributions inferred from the accepted sample as the estimator of the parameter, and one using the median of the same distribution. Three sets of PODs were carried out, each for a different scenario, with a different number of parameters and complexity (figure 2): the first scenario contained only one statistical group, five summary statistics and three unknown parameters. The second example contained 19 summary statistics from three statistical groups and had five unknown parameters, while the third example had 18 summary statistics, three statistical groups and seven parameters; the last two examples included heterochronous samples (see boxes S1-S9 in supplementary material for the input files of these scenarios for simulations in the programs mentioned below, and table S3 for a summary of the summary statistics employed in the performance analysis).

In order to provide a reference for the qualitative and performance evaluations, the analyses were carried out in parallel with two other softwares: the program BSSC [40] complemented with the abc package [37] designed to run in the R environment [42] hence abbreviated BSSC+Rabc, and the program DIYABC [38].

## Results and Discussion

### Qualitative comparison with other software

Table 1 shows a summary of chosen features of BaySICS and two other software alternatives. No software has all the desirable features, even for a single category out of the three evaluated (interface, simulations, post-simulation analysis). The parameters estimation and model choice analyses resulted in reasonably similar inferences by the three softwares. The output of the analyses is shown in figure S2 and table S2. In a subjective account of features and their importance, we found BaySICS to have a good balance of features that complement those of the other software alternatives without a serious deficiency in any category.

**Table 1:** Comparison of chosen features of the three software options.

	BaySICS	BSSC + R-ABC	DIYABC
***Interface***			
Integrated platform	Yes	No	Yes
Graphic interface	Yes	No	Yes
***Coalescent Simulations***			
Heterochronous sampling (e.g. ancient DNA)	Yes	Yes	Yes
Flexible statistical grouping	Very good	Good	No
Number of summary statistics (DNA)^1^	7/2/4	7/4/1	8/5/0
Population split	Yes	Yes	Yes
Populations merging	Yes	No	Yes
*N_e_* change	Yes	Yes	Yes
Exponential growth	Yes	Yes	No
Gene flow	Yes	Yes	No
User specified formulas	No	Yes	No
Input commands^2^	Yes	No	No
Cross reference of priors	Yes	Yes/No^3^	Yes/No^4^
Multilocus data	No	No	Yes
DNA sequence data	Yes	Yes	Yes
Other markers (microsatellites, SNP's)	No	Yes	Yes
Number of available prior distributions	6	5	4
Time^5^ to complete 1 million simulations	3.06	13.10	2.22
***Analysis***			
Number of algorithms for rejection	2	1	1
Adjustment algorithms (regression)	1	2	2
Transformation of variables	No	Yes	Yes
Model choice analysis	Yes	Yes	Yes
Algorithms for model choice	2	1	2
Charts of joint posteriors	Yes	Yes/No^6^	-
Charts of accepted summary statistics	Yes	Yes	-^7^
***Others***			
MCMC without likelihoods	Yes	No	No
Validation analyses^8^	Yes	Yes	Yes

Notes:

### Theoretical validation and performance

For all the scenarios tested, which by necessity were simple (one/two populations, one event), the values of *T_MRCA_* and tree lengths, as averaged from 10 000 simulations, matched the expected values with a 1% accuracy (table S1). Also the estimated summary statistics were reasonably similar to those calculated with Arlequin (data not shown).

The test for simulation speed gave somewhat conflicting results. For models with low mutation rates, BaySICS had a similar speed to DIYABC, which was up to 7–10 times faster than BSSC, but for models with high mutation rates (>0.5 mutations per nucleotide site per million years; /base/10^6^y) BaySICS became slower than BSSC. This difference could be attributed to the assignment of mutations with heterogeneous rates among sites, for which BaySICS assigns mutations one by one. If extremely high mutation rates need to be tested in BaySICS, we therefore recommend to run in parallel many short runs or to use MCMCwL.

The assessment of performance using MCMCwL resulted in accurate inference of posterior distributions when compared with the posteriors obtained from the ordinary analysis (data not shown), with only slight gains in computational effort. However, a real improvement was observed in enabling a way to perform analysis that would be too long and exhaustive if performed in an ordinary way. For instance, running scenario D in figure S1, in parallel and with MCMCwL yielded a set of reference tables with a total of 10 million simulations in 24 hours in a desktop computer. When coupled with a further rejection step, the analysis yielded a posterior sample of 10,000 simulations with an approaching error of zero for some summary statistics (as haplotypes number). An equivalent analysis without MCMCwL would have required one billion simulations and considerably longer processing time.

Table 2 shows the performance measures (mean relative bias, RRMSE, 50% and 95% coverage and factor 2) obtained by using both median and mode as the punctual estimators of the parameters for the simulated example 1 (figure 2). Tables S4 and S5 show the equivalent measures for the simulated examples 2 and 3. Despite presenting some differences that could be attributed to technical issues in the software comparison (see table S5), the three softwares provided remarkably similar standards of performance. Both accuracy (as measured by relative bias, RRMSE and factor 2) and precision (as shown by coverage values) showed reasonable behaviour.

**Table 2:** Measures of performance for parameters estimation for the simulated example 1 performed by the three software alternatives.

Parameters	BaySICS		BSSC + Rabc	DIYABC	
Statistics	Mode	Median	Median	Mode	Median
***N_e1_***					
*Relative bias*	0.0050	0.0697	0.0501	0.0077	0.0693
*RRMSE*	0.3207	0.3503	0.3269	0.3390	0.3610
*Coverage 50/95*	0.5155	0.9661	-	0.5040	0.9570
*Factor 2*	0.9581	0.9673	0.9676	0.9470	0.9630
***t***					
*Relative bias*	0.0907	0.1401	0.1706	0.0483	0.1728
*RRMSE*	0.5549	0.5652	0.6671	0.6220	0.6660
*Coverage 50/95*	0.5216	0.9632	-	0.4900	0.9530
*Factor 2*	0.8795	0.9087	0.8919	0.8360	0.8770
***N_e2_***					
*Relative bias*	0.0767	0.2624	0.3455	0.3048	0.3773
*RRMSE*	0.8051	0.8412	0.9938	1.2860	1.0730
*Coverage 50/95*	0.5168	0.9609	-	0.5090	0.9490
*Factor 2*	0.6969	0.8219	0.7976	0.5980	0.8050

The two values displayed for coverage correspond to the coverage of 50% (left) and 95% (right) and not to the values corresponding to mode and median (coverage does not depend on the punctual estimation).

### Features of BaySICS

**Figure 3:**
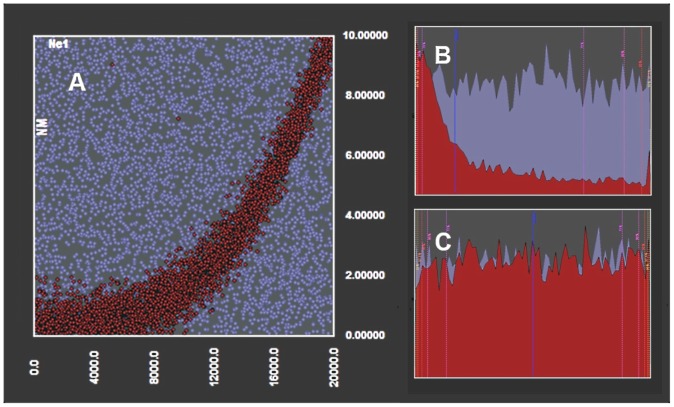
Joint posterior distribution of two hypothetical parameters as displayed by BaySICS. The area or points in red represent the approached posterior distributions conditioned to the observed summary statistics; while the purple area or points represent the prior distributions. A) The joint posterior of hypothetical parameters *NM* and *Ne*1 is quite informative since the region of high likelihood is narrow and well differentiated from the prior. This interpretation would not be possible by looking only at the single-dimensional posteriors. B) The posterior for parameter *NM*. C) The posterior of *Ne*1, where the posterior does not differ from the prior, giving the false perception that rejection (i.e. conditioning to the observed data) has no effect for this parameter. This example was run by an artificial reference table but the shape of the posterior was inspired in a real case study.

Besides performance, the contribution of a software to the community of potential users relies on the features that are not shared with other software alternatives. The set of exclusive features implemented in BaySICS, vary from being just time-saving to playing a more fundamental role in the assessment of conclusions. The automatic calibration of radiocarbon dates is an example of a purely time-saving feature. The flexible definition of statistical groups allows alternative groupings of samples in, for example, scenarios with complicated population structure, which can reduce excessive use of summary statistics with the concurrent improvement in approaching error. One of the most relevant features is the display of joint posterior distributions in 2-D or 3-D charts. The utility of this feature relies on the fact that while ordinary single-dimensional posteriors may seem uninformative, joint posterior distributions can produce very informative displays that would instead require the analysis of combinations of parameter values rather than individual ones. As shown in figure 3, some regions with specific combinations of parameter values could be significantly more likely than other regions, a pattern that can only be assessed by a joint posterior. Furthermore, even posteriors that are unimodal and bell-shaped may be better interpreted in a joint posterior as the one in figure 3 B.

The number of potential theoretical contributions for improving accuracy and efficiency in ABC analysis is large and increasing. Selection of proper implementations is a demanding task, since theoretical expectations cannot always be reached in empirical applications. In addition, not all analyses included in many programs prove useful or pertinent for the scope of many studies. For these reasons, BaySICS provides a basic toolbox for performing inference from simulations based on ABC, and includes additional features that are useful for ancient DNA studies. These features render BaySICS applicable in a wide range of studies, besides those with heterochronous data. BaySICS can be downloaded at: https://www.dropbox.com/sh/6wsh252lsiizhri/hGy04j4tbD.

## Supporting Information

Figure S1 **Simulated examples employed for the qualitative evaluation.** A) simulated scenario employed to perform a parameter estimation analysis. It contains nine parameters: effective population sizes of the present populations (*N_e_*_1_, *N_e_*_2_ and *N_e_*_3_), as well as the ancestral populations (*N_e_*_4_, *N_e_*_5_ and *N_e_*_6_), the time to the demographic expansion of population 2 (*t_1_*) and the time to two split events (*t*_2_ and *t*_3_). Real values were *N_e_*_1_ = 10 000, *N_e_*_2_ = 15 000, *N_e_*_3_ = 5 000, *N_e_*_4_ = 2 814, *N_e_*_5_ = 17 814, *N_e_*_6_ = 6 284, *t_1_* = 5 000, *t*_2_ = 10 000, and *t*_3_ = 40 000. B-E) four competing models that were used for model choice analysis (where third model -bottleneck- was the right one). In the scenario for parameters estimation (A), the generation time was one per year, the mutation rate was set to 0.15 per site per million years (/site/10^6^y), the transition/transversion bias was 0.875 and the gamma shape parameter was 0.15. The sample consisted of 51 heterochronous DNA sequences with ages ranging from 0–38 910 years before present, and DNA sequences were 1 000 bp long. In the scenarios for model choice (B-E) the generation time was 15 years, the mutation rate was set to 0.247/site/10^6^y, the transition/transversion bias was 0.9798 and the gamma shape parameter was 0.05. The sample consisted of 59 heterochronous DNA sequences with ages ranging from 3 685 to 61 600 years before present. Analyzed sequences were 741 bp long. Both analyses were inspired by real data from case studies of ancient DNA.
(DOCX)

Figure S2 **Posterior distributions of parameters estimated from the simulated data with three software options.** Parameter names correspond to the ones in figure S1A. Vertical scales represent probability but their interpretation is relative so they were removed. Prior distributions are indicated as grey dotted lines in charts from BSSC+Rabc and BaySICS and a red line in DIYABC. The red curve indicates the posterior inferred from linear regression while the black curve indicates the posterior inferred from simple rejection in BSSC+Rabc. Small colorful graphs are charts obtained in BaySICS with the options ‘histogram’ and ‘color informative’. Vertical dotted lines in purple represent the real value. Note that this charts are not intended for any performance assesment but only to make a qualitative comparison of the programs' output. Charts were distorted to match their horizontal scales.
(DOCX)

Table S1 Expected and observed mean heights and lengths of the coalescent trees for 10 test examples. The expected values for the time to the most recent common ancestor (*T_MRCA_*) and tree length (*L*) were obtained by formulas (theoretical) while the observed ones were obtained by averaging over 10 000 simulations that were performed with the programs BaySICS and Bayesian Serial Simcoal (in the case of BSSC, only *T_MRCA_*). The test scenarios corresponded to: 1–3) consisted of a single population with a single sample consisting of contemporary samples and no demographic events; 4–6) consisted of a single population with samples taken at two times, one at generation zero and one at the generation *T* indicated in the parameters column. In addition, the population was subject to a sudden change of effective population size going from size *N_e_*_1_ to size *N_e_*_2_. The time (in generations) of this change coincided exactly with the time of sampling of the older sample; 7–10) consisted of two populations with sizes *N_e_*_1_ and *N_e_*_2_ that originated from a split event that happened *T* generations ago from an ancestral population with size *N_e_*_3_. Two contemporary samples were taken, one from each present population. For examples 4–10, the theoretical expectation was obtained by adding the expected lengths of each population sub-tree or each sub-tree obtained from one contemporary subsample plus the length of the lineages between the local MRCA and the next event. Shown examples correspond only to a subset of the examples analyzed. Additional tests involved different sample sizes and parameter values as well as times to coalescent events other than the MRCA. Notice that tests for different values of *N_e_* are somehow redundant since *L* and *T_MRCA_* only depend on *N_e_* as a scaling factor; unless heterochronous samples or demographic events are involved.
(DOCX)

Table S2 Bayes factors among all pairwise comparisons of the models tested in the model comparison analysis. They correspond to scenarios B-E of figure S1. The rows in bold indicate the model that obtained the highest support. Notice that this is just an essay for a qualitative evaluation of the differences among programs and not a test of performance.
(DOCX)

Table S3 Summary statistics employed for the performance test of parameters estimation analysis. Abbreviations of the summary statistics mean: *HapTypesX*: number of haplotypes in statistical group X; *PrivHapsX*: number of private haplotypes in statistical group X; *SegSitesX*: number of segregating sites in statistical group X; *PrivSegX*: number of private segregating sites in statistical group X; *NucDiverX*: nucleotide diversity in statistical group X; *PairDiffsX*: average number of pairwise differences in statistical group X; *GenDiverX*: gene diversity in statistical group X; *VarPairD*: variance of pairwise differences; *PairDiffsXvsY*: average number of pairwise differences between statistical group X and statistical group Y; *FstXvsY*: F_ST_ between statistical groups X and Y. Since nucleotide and average number of pairwise differences carry practically the same information (NucDiver = PairDiffs/n; n = number of nucleotides), they are set in the same cell. Since the sets of summary statistics available for each program were different, a set that differed at minimum were chosen; the rationale was that using identical sets of summary statistics would be pointless since the differences among programs include the set of summary statistics they have implemented.
(DOCX)

Table S4 Measures of performance for parameters estimation for the simulated example 2. The two values displayed for coverage correspond to the coverage of 50% (left) and 95% (right) and not to the values corresponding to mode and median (coverage does not depend of the punctual estimation).
(DOCX)

Table S5 Measures of performance for parameters estimation for the simulated example 3. The two values displayed for coverage correspond to the coverage of 50% (left) and 95% (right) and not to the values corresponding to mode and median, (coverage does not depend of the punctual estimation). In both simulated example 2 and simulated example 3, the estimates obtained with DIYABC presented important inconsistencies when the number of iterations (PODs) varied: the ones obtained for 10 000 iterations were one order of magnitude larger than those obtained with 1 000 iterations. In addition, most of the values are much more similar between BSSC+Rabc and BaySICS. Those facts could be indicative of a numerical issue of the type of “catastrophic cancelation”. So the true performance measures for DIYABC clearly should be much better for those parameters.
(DOCX)

Box S1 **Input file for simulation of the Simulated Example 1 in BaySICS.**
(DOCX)

Box S2 **Input file for simulation of the Simulated Example 2 in BaySICS.**
(DOCX)

Box S3 **Input file for simulation of the Simulated Example 3 in BaySICS.**
(DOCX)

Box S4 **Input file for simulation of the Simulated Example 1 in BSSC.**
(DOCX)

Box S5 **Input file for simulation of the Simulated Example 2 in BSSC.**
(DOCX)

Box S6 **Input file for simulation of the Simulated Example 3 in BSSC.**
(DOCX)

Box S7 **Header file for simulation of the Simulated Example 1 in DIYABC.**
(DOCX)

Box S8 **Header file for simulation of the Simulated Example 2 in DIYABC.**
(DOCX)

Box S9 **Header file for simulation of the Simulated Example 3 in DIYABC.**
(DOCX)
