# Supplementary material for: Back to BaySICS: A User-Friendly Program for Bayesian Statistical Inference from Coalescent Simulations
Source: PLoS One. 2014 May 27;9(5):e98011. doi: 10.1371/journal.pone.0098011 (PMC4035278; doi:10.1371/journal.pone.0098011)
Supplement: Figure S1 — Simulated examples employed for the qualitative evaluation. A) simulated scenario employed to perform a parameter estimation analysis. It contains nine parameters: effective population sizes of the present populations (Ne 1, Ne 2 and Ne 3), as well as the ancestral populations (Ne 4, Ne 5 and Ne 6), the time to the demographic expansion of population 2 (t1) and the time to two split events (t 2 and t 3). Real values were Ne 1 = 10 000, Ne 2 = 15 000, Ne 3 = 5 000, Ne 4 = 2 814, Ne 5 = 17 814, Ne 6 = 6 284, t1 = 5 000, t 2 = 10 000, and t 3 = 40 000. B-E) four competing models that were used for model choice analysis (where third model -bottleneck- was the right one). In the scenario for parameters estimation (A), the generation time was one per year, the mutation rate was set to 0.15 per site per million years (/site/106y), the transition/transversion bias was 0.875 and the gamma shape parameter was 0.15. The sample consisted of 51 heterochronous DNA sequences with ages ranging from 0–38 910 years before present, and DNA sequences were 1 000 bp long. In the scenarios for model choice (B-E) the generation time was 15 years, the mutation rate was set to 0.247/site/106y, the transition/transversion bias was 0.9798 and the gamma shape parameter was 0.05. The sample consisted of 59 heterochronous DNA sequences with ages ranging from 3 685 to 61 600 years before present. Analyzed sequences were 741 bp long. Both analyses were inspired by real data from case studies of ancient DNA. (DOCX) [file pone.0098011.s001.docx]

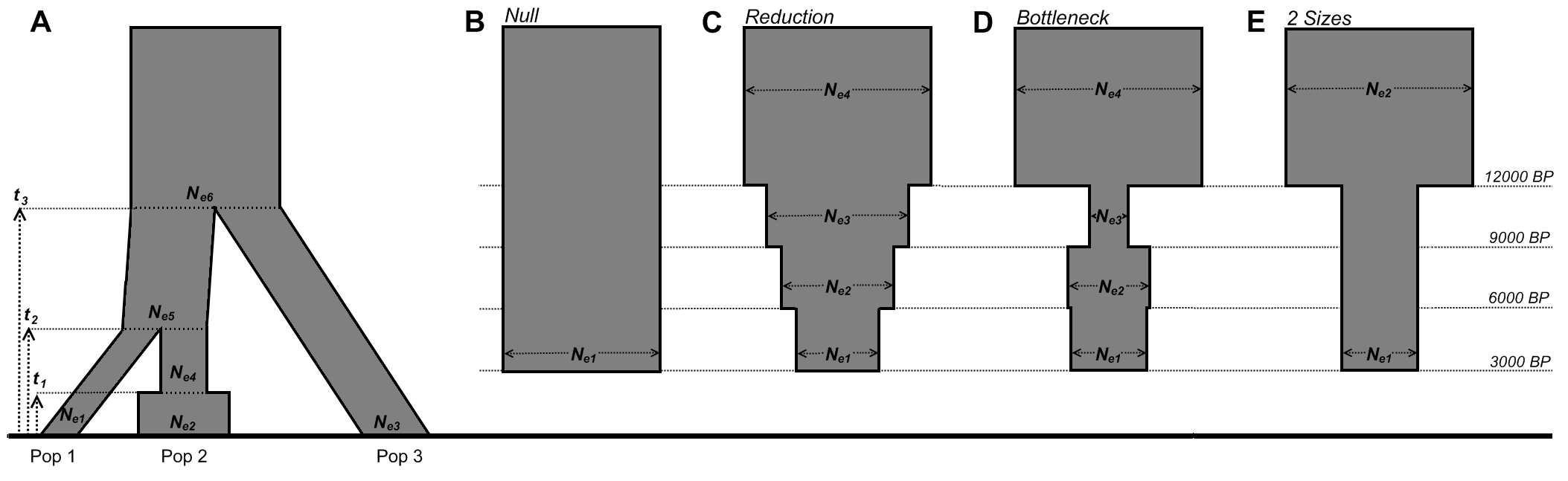


**Figure SF 1. Simulated examples employed for the qualitative evaluation.** A) simulated scenario employed to perform a parameter estimation analysis. It contains nine parameters: effective population sizes of the present populations (*N_e_*_1_, *N_e_*_2_ and *N_e_*_3_), as well as the ancestral populations (*N_e_*_4_, *N_e_*_5_ and *N_e_*_6_), the time to the demographic expansion of population 2 (*t_1_*) and the time to two split events (*t*_2_ and *t*_3_). Real values were *N_e_*_1_ = 10 000, *N_e_*_2_ = 15 000, *N_e_*_3_ = 5 000, *N_e_*_4_ = 2 814, *N_e_*_5_ = 17 814, *N_e_*_6_ = 6 284, *t_1_* = 5 000, *t*_2_ = 10 000, and *t*_3_ = 40 000. B-E) four competing models that were used for model choice analysis (where third model -bottleneck- was the right one). In the scenario for parameters estimation (A), the generation time was one per year, the mutation rate was set to 0.15 per site per million years (/site/10^6^y), the transition/transversion bias was 0.875 and the gamma shape parameter was 0.15. The sample consisted of 51 heterochronous DNA sequences with ages ranging from 0 - 38 910 years before present, and DNA sequences were 1 000 bp long. In the scenarios for model choice (B - E) the generation time was 15 years, the mutation rate was set to 0.247 /site/10^6^y , the transition/transversion bias was 0.9798 and the gamma shape parameter was 0.05. The sample consisted of 59 heterochronous DNA sequences with ages ranging from 3 685 to 61 600 years before present. Analyzed sequences were 741 bp long. Both analyses were inspired by real data from case studies of ancient DNA.
