# Supplementary material for: Back to BaySICS: A User-Friendly Program for Bayesian Statistical Inference from Coalescent Simulations
Source: PLoS One. 2014 May 27;9(5):e98011. doi: 10.1371/journal.pone.0098011 (PMC4035278; doi:10.1371/journal.pone.0098011)
Supplement: Figure S2 — Posterior distributions of parameters estimated from the simulated data with three software options. Parameter names correspond to the ones in figure S1A. Vertical scales represent probability but their interpretation is relative so they were removed. Prior distributions are indicated as grey dotted lines in charts from BSSC+Rabc and BaySICS and a red line in DIYABC. The red curve indicates the posterior inferred from linear regression while the black curve indicates the posterior inferred from simple rejection in BSSC+Rabc. Small colorful graphs are charts obtained in BaySICS with the options ‘histogram’ and ‘color informative’. Vertical dotted lines in purple represent the real value. Note that this charts are not intended for any performance assesment but only to make a qualitative comparison of the programs' output. Charts were distorted to match their horizontal scales. (DOCX) [file pone.0098011.s002.docx]

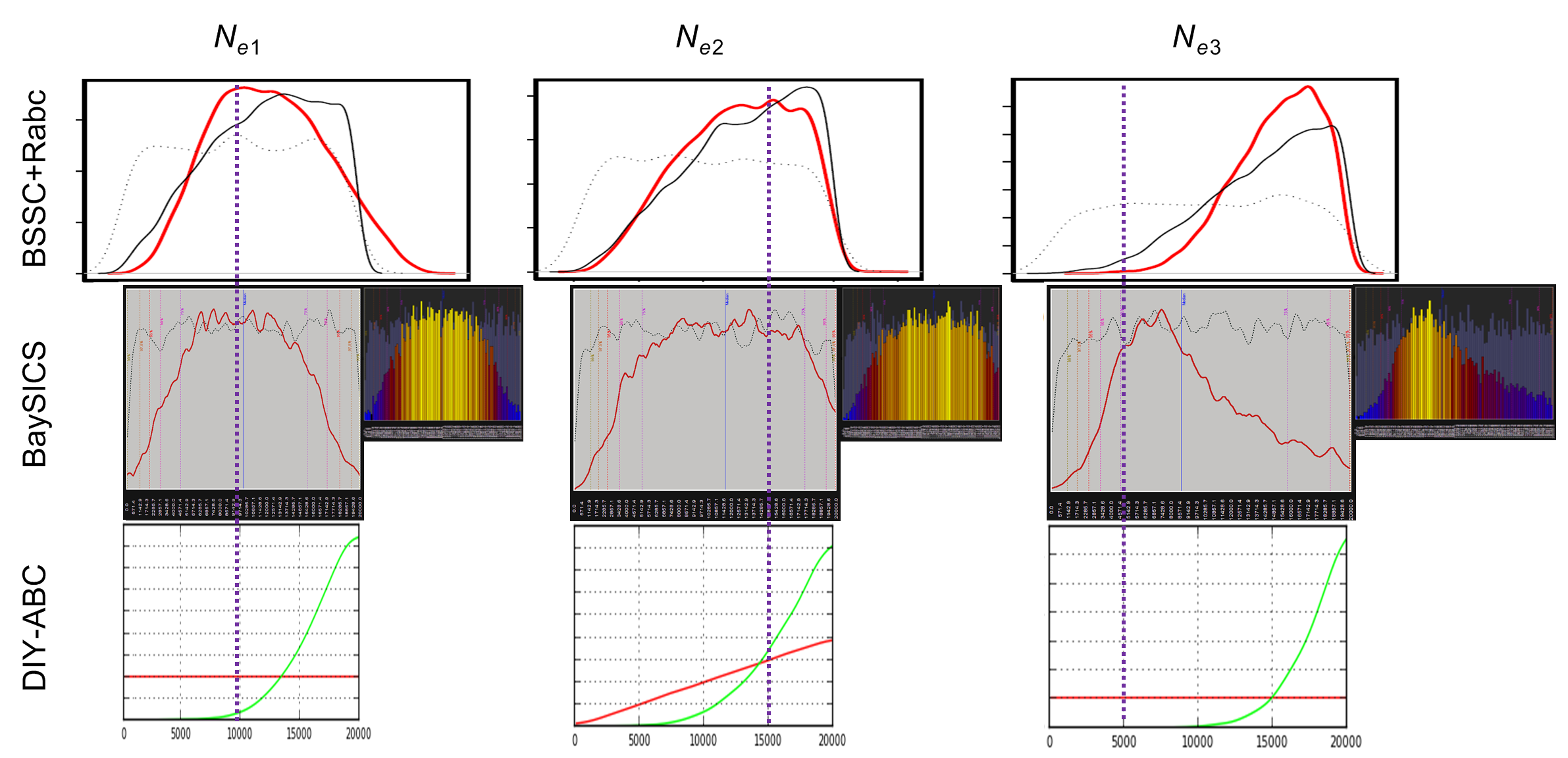


**Figure SF 2. Posterior distributions of parameters estimated from the simulated data with three software options.** Parameter names correspond to the ones in figure SF 1A. Vertical scales represent probability but their interpretation is relative so they were removed. Prior distributions are indicated as grey dotted lines in charts from BSSC+Rabc and BaySICS and a red line in DIYABC. The red curve indicates the posterior inferred from linear regression while the black curve indicates the posterior inferred from simple rejection in BSSC+Rabc. Small colorful graphs are charts obtained in BaySICS with the options ‘histogram’ and ‘color informative’. Vertical dotted lines in purple represent the real value. Note that this charts are not intended for any performance assesment but only to make a qualitative comparison of the programs’ output. Charts were distorted to match their horizontal scales.


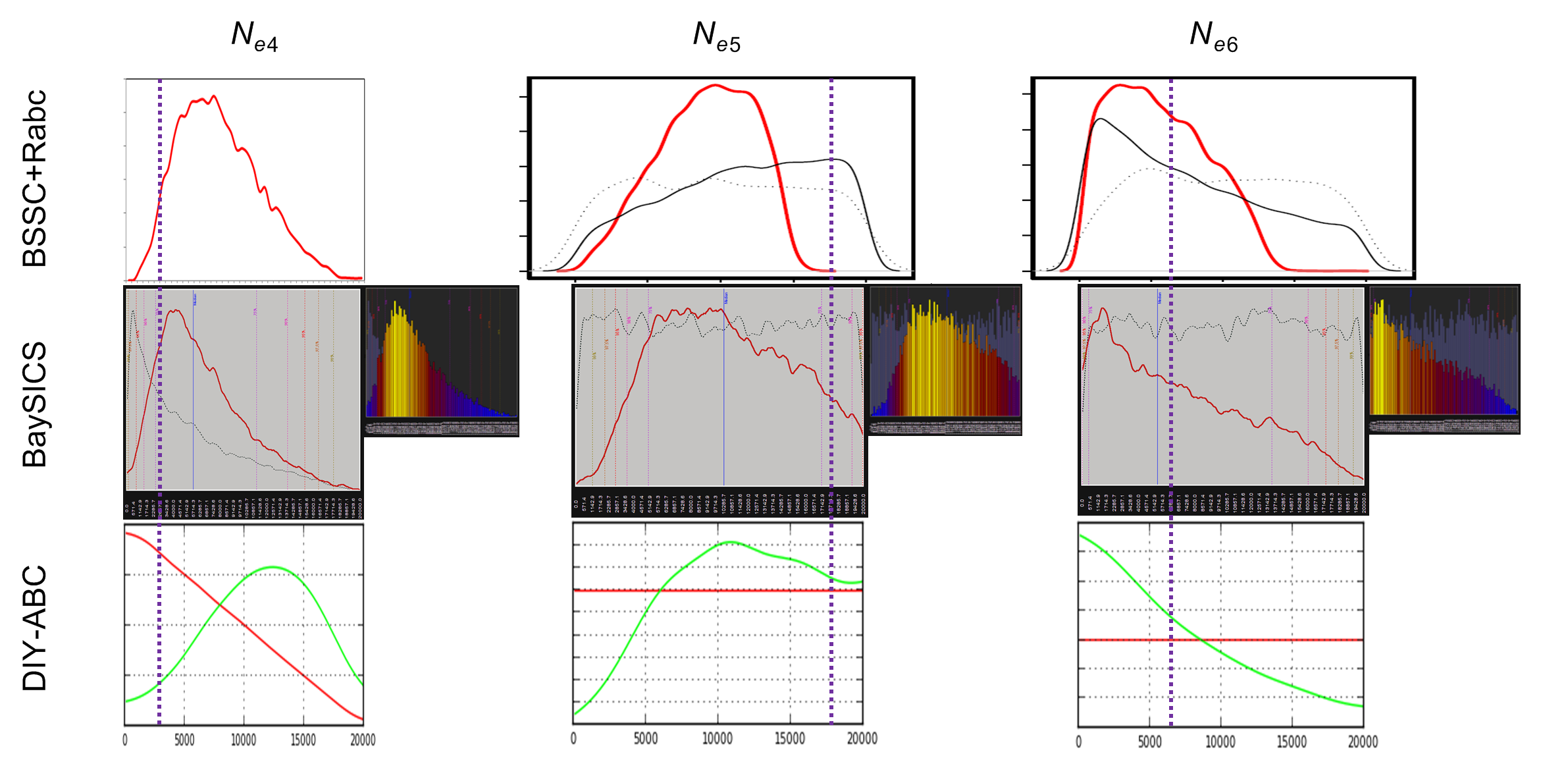


Figure SF 2 (continued). Posterior distributions of parameters estimated from the simulated data with three software options.


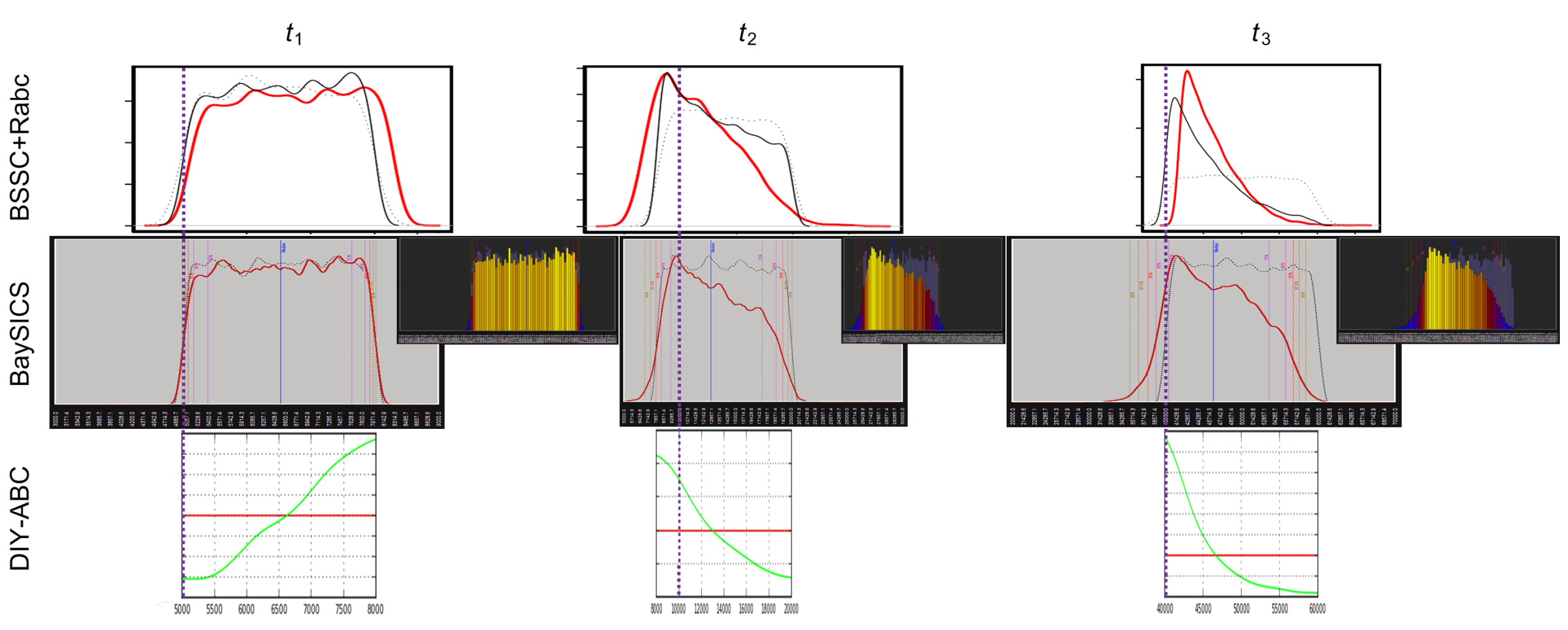


Figure SF 2 (continued). Posterior distributions of parameters estimated from the simulated data with three software options.
