# Supplementary material for: Back to BaySICS: A User-Friendly Program for Bayesian Statistical Inference from Coalescent Simulations
Source: PLoS One. 2014 May 27;9(5):e98011. doi: 10.1371/journal.pone.0098011 (PMC4035278; doi:10.1371/journal.pone.0098011)
Supplement: Table S1 — Expected and observed mean heights and lengths of the coalescent trees for 10 test examples. The expected values for the time to the most recent common ancestor (TMRCA) and tree length (L) were obtained by formulas (theoretical) while the observed ones were obtained by averaging over 10 000 simulations that were performed with the programs BaySICS and Bayesian Serial Simcoal (in the case of BSSC, only TMRCA). The test scenarios corresponded to: 1–3) consisted of a single population with a single sample consisting of contemporary samples and no demographic events; 4–6) consisted of a single population with samples taken at two times, one at generation zero and one at the generation T indicated in the parameters column. In addition, the population was subject to a sudden change of effective population size going from size Ne 1 to size Ne 2. The time (in generations) of this change coincided exactly with the time of sampling of the older sample; 7–10) consisted of two populations with sizes Ne 1 and Ne 2 that originated from a split event that happened T generations ago from an ancestral population with size Ne 3. Two contemporary samples were taken, one from each present population. For examples 4–10, the theoretical expectation was obtained by adding the expected lengths of each population sub-tree or each sub-tree obtained from one contemporary subsample plus the length of the lineages between the local MRCA and the next event. Shown examples correspond only to a subset of the examples analyzed. Additional tests involved different sample sizes and parameter values as well as times to coalescent events other than the MRCA. Notice that tests for different values of Ne are somehow redundant since L and TMRCA only depend on Ne as a scaling factor; unless heterochronous samples or demographic events are involved. (DOCX) [file pone.0098011.s003.docx]

**Table ST 1. Expected and observed mean heights and lengths of the coalescent trees for 10 test examples.** The expected values for the time to the most recent common ancestor (*T_MRCA_*) and tree length (*L*) were obtained by formulas (theoretical) while the observed ones were obtained by averaging over 10 000 simulations that were performed with the programs BaySICS and Bayesian Serial Simcoal (in the case of BSSC, only *T_MRCA_*). The test scenarios corresponded to: 1-3) consisted of a single population with a single sample consisting of contemporary samples and no demographic events; 4-6) consisted of a single population with samples taken at two times, one at generation zero and one at the generation *T* indicated in the parameters column. In addition, the population was subject to a sudden change of effective population size going from size *N_e_*_1_ to size *N_e_*_2_. The time (in generations) of this change coincided exactly with the time of sampling of the older sample; 7-10) consisted of two populations with sizes *N_e_*_1_ and *N_e_*_2_ that originated from a split event that happened *T* generations ago from an ancestral population with size *N_e_*_3_. Two contemporary samples were taken, one from each present population. For examples 4-10, the theoretical expectation was obtained by adding the expected lengths of each population sub-tree or each sub-tree obtained from one contemporary subsample plus the length of the lineages between the local MRCA and the next event. Shown examples correspond only to a subset of the examples analyzed. Additional tests involved different sample sizes and parameter values as well as times to coalescent events other than the MRCA. Notice that tests for different values of *N_e_* are somehow redundant since *L* and *T_MRCA_* only depend on *N_e_* as a scaling factor; unless heterochronous samples or demographic events are involved.

| # | Parameters | Sample size | Theoretical | | BaySICS | | BSSC |
| --- | --- | --- | --- | --- | --- | --- | --- |
|  | *N_e_*_1_, *N_e_*_2_, *N_e3_* / *T* |  | *E*(*T_MRCA_*) | *E*(*L*) | *T_MRCA_* | *L* | *T_MRCA_* |
| 1 | 100 | 100 | 198.0 | 1035.4 | 199.2 | 1036.4 | 182.75 |
| 2 | 25000 | 100 | 49500.0 | 258868.9 | 49692.3 | 259189.3 | 49366.8 |
| 3 | 135000 | 100 | 267300.0 | 1397891.9 | 268163.84 | 1396814.5 | 270368.6 |
| 4 | 100, 1000 / 1000 | 50+50 | 2960.8 | 10741.5 | 2947.22 | 10675.3 | 2938.8 |
| 5 | 250, 5000 / 2500 | 50+50 | 12303.9 | 49447.7 | 12295.4 | 49089.8 | 12177.8 |
| 6 | 1000, 75400 / 34500 | 50+50 | 182343.1 | 722975.4 | 181174.4 | 713456.8 | 181964.9 |
| 7 | 432, 625, 345 / 2350 | 50/50 | 2695.0 | 12829.6 | 2702.5 | 12760.6 | 2704.5 |
| 8 | 125, 250, 3450 / 1250 | 50/50 | 4700.0 | 12039.4 | 4689.5 | 12013.3 | 4729.6 |
| 9 | 1285, 17500, 6300 / 53500 | 50/50 | 59800.0 | 251816.5 | 60157.1 | 249302.9 | 60133.1 |
| 10 | 15800, 27500, 42000 / 71000 | 50/50 | 113000.0 | 530763.2 | 116735.3 | 532953.0 | 116117.1 |
