# Supplementary material for: Back to BaySICS: A User-Friendly Program for Bayesian Statistical Inference from Coalescent Simulations
Source: PLoS One. 2014 May 27;9(5):e98011. doi: 10.1371/journal.pone.0098011 (PMC4035278; doi:10.1371/journal.pone.0098011)
Supplement: Table S2 — Bayes factors among all pairwise comparisons of the models tested in the model comparison analysis. They correspond to scenarios B-E of figure S1. The rows in bold indicate the model that obtained the highest support. Notice that this is just an essay for a qualitative evaluation of the differences among programs and not a test of performance. (DOCX) [file pone.0098011.s004.docx]

**Table ST 2. Bayes factors among all pairwise comparisons of the models tested in the model comparison analysis.** They correspond to scenarios B-E of figure SF 1. The rows in bold indicate the model that obtained the highest support. Notice that this is just an essay for a qualitative evaluation of the differences among programs and not a test of performance.

|  |  | |  | |  | |  | |
| --- | --- | --- | --- | --- | --- | --- | --- | --- |
| *BaySICS* | | *Null* | | *Reduction* | | *Bottleneck* | | *2-sizes* |
| *Relative support* | | 0.0094 | | 0.0003 | | 0.0158 | | 0.0135 |
| *Null* | | 1.000 | | 13.847 | | 0.468 | | 0.532 |
| *Reduction* | | 0.072 | | 1.000 | | 0.062 | | 0.068 |
| ***Bottleneck*** | | **2.137** | | **16.129** | | **1.000** | | **1.170** |
| *2-sizes* | | 1.879 | | 14.705 | | 0.850 | | 1.000 |
|  | |  | |  | |  | |  |
| *BSSC+Rabc* | | *Null* | | *Reduction* | | *Bottleneck* | | *2-sizes* |
| *Relative support* | | 0.0143 | | 0.0315 | | 0.9542 | | 0.0000 |
| *Null* | | 1.000 | | 0.454 | | 0.015 | | 453.78 |
| *Reduction* | | 2.205 | | 1.000 | | 0.033 | | 1000.5 |
| ***Bottleneck*** | | **66.849** | | **30.321** | | **1.000** | | **30335** |
| *2-sizes* | | 0.002 | | 0.001 | | 0.000 | | 1.000 |
|  | |  | |  | |  | |  |
| *DIY-ABC* | | *Null* | | *Reduction* | | *Bottleneck* | | *2-sizes* |
| *Relative support* | | 0.0025 | | 0.0873 | | 0.3632 | | 0.5471 |
| *Null* | | 1.000 | | 0.029 | | 0.007 | | 0.005 |
| *Reduction* | | 34.920 | | 1.000 | | 0.240 | | 0.160 |
| *Bottleneck* | | 145.28 | | 4.160 | | 1.000 | | 0.664 |
| ***2-sizes*** | | **218.84** | | **6.267** | | **1.506** | | **1.000** |
