# Supplementary material for: Back to BaySICS: A User-Friendly Program for Bayesian Statistical Inference from Coalescent Simulations
Source: PLoS One. 2014 May 27;9(5):e98011. doi: 10.1371/journal.pone.0098011 (PMC4035278; doi:10.1371/journal.pone.0098011)
Supplement: Table S3 — Summary statistics employed for the performance test of parameters estimation analysis. Abbreviations of the summary statistics mean: HapTypesX: number of haplotypes in statistical group X; PrivHapsX: number of private haplotypes in statistical group X; SegSitesX: number of segregating sites in statistical group X; PrivSegX: number of private segregating sites in statistical group X; NucDiverX: nucleotide diversity in statistical group X; PairDiffsX: average number of pairwise differences in statistical group X; GenDiverX: gene diversity in statistical group X; VarPairD: variance of pairwise differences; PairDiffsXvsY: average number of pairwise differences between statistical group X and statistical group Y; FstXvsY: FST between statistical groups X and Y. Since nucleotide and average number of pairwise differences carry practically the same information (NucDiver = PairDiffs/n; n = number of nucleotides), they are set in the same cell. Since the sets of summary statistics available for each program were different, a set that differed at minimum were chosen; the rationale was that using identical sets of summary statistics would be pointless since the differences among programs include the set of summary statistics they have implemented. (DOCX) [file pone.0098011.s005.docx]

**Table ST 3. Summary statistics employed for the performance test of parameters estimation analysis.** Abbreviations of the summary statistics mean: *HapTypesX*: number of haplotypes in statistical group X; *PrivHapsX*: number of private haplotypes in statistical group X; *SegSitesX*: number of segregating sites in statistical group X; *PrivSegX*: number of private segregating sites in statistical group X; *NucDiverX*: nucleotide diversity in statistical group X; *PairDiffsX*: average number of pairwise differences in statistical group X; *GenDiverX*: gene diversity in statistical group X; *VarPairD*: variance of pairwise differences; *PairDiffsXvsY*: average number of pairwise differences between statistical group X and statistical group Y; *FstXvsY*: F_ST_ between statistical groups X and Y. Since nucleotide and average number of pairwise differences carry practically the same information (NucDiver=PairDiffs/n; n=number of nucleotides), they are set in the same cell. Since the sets of summary statistics available for each program were different, a set that differed at minimum were chosen; the rationale was that using identical sets of summary statistics would be pointless since the differences among programs include the set of summary statistics they have implemented.

|  | *Simulated Example 1* | | | *Simulated Example 2* | | | *Simulated Example 3* | | |
| --- | --- | --- | --- | --- | --- | --- | --- | --- | --- |
|  | ***BaySICS*** | ***BSSC*** | ***DIYABC*** | ***BaySICS*** | ***BSSC*** | ***DIYABC*** | ***BaySICS*** | ***BSSC*** | ***DIYABC*** |
| *HapTypes1* | × | × | × | × | × | × | × | × | × |
| *PrivHaps1* |  |  |  | × | × |  |  |  |  |
| *SegSites1* | × | × | × | × | × | × | × | × | × |
| *PrivSeg1* |  |  |  |  |  | × |  |  |  |
| *NucDiver1/PairDiffs1* | × | × | × | × | × | × | × | × | × |
| *GenDiver1* | × | × |  |  |  |  |  |  |  |
| *VarPairD1* |  |  | × |  |  |  |  |  |  |
| *TajimasD1* | × | × | × | × | × | × | × | × | × |
| *HapTypes2* |  |  |  | × | × | × | × | × | × |
| *PrivHaps2* |  |  |  | × | × |  |  |  |  |
| *SegSites2* |  |  |  | × | × | × | × | × | × |
| *PrivSeg2* |  |  |  |  |  | × |  |  |  |
| *NucDiver2/PairDiffs2* |  |  |  | × | × | × | × | × | × |
| *TajimasD2* |  |  |  | × | × | × | × | × | × |
| *HapTypes3* |  |  |  | × | × | × | × | × | × |
| *PrivHaps3* |  |  |  | × | × |  |  |  |  |
| *SegSites3* |  |  |  | × | × | × | × | × | × |
| *PrivSeg3* |  |  |  |  |  | × |  |  |  |
| *NucDiver3/PairDiffs3* |  |  |  | × | × | × | × | × | × |
| *TajimasD3* |  |  |  | × | × | × | × | × | × |
| *PairDiffs1vs2* |  |  |  | × | × | × | × | × | × |
| *Fst1vs2* |  |  |  | × | × | × | × | × | × |
| *PairDiffs1vs3* |  |  |  |  |  |  | × | × | × |
| *Fst1vs3* |  |  |  |  |  |  | × | × | × |
| *PairDiffs2vs3* |  |  |  | × | × | × | × | × | × |
| *Fst2vs3* |  |  |  | × | × | × | × | × | × |
