# Supplementary material for: Back to BaySICS: A User-Friendly Program for Bayesian Statistical Inference from Coalescent Simulations
Source: PLoS One. 2014 May 27;9(5):e98011. doi: 10.1371/journal.pone.0098011 (PMC4035278; doi:10.1371/journal.pone.0098011)
Supplement: Table S4 — Measures of performance for parameters estimation for the simulated example 2. The two values displayed for coverage correspond to the coverage of 50% (left) and 95% (right) and not to the values corresponding to mode and median (coverage does not depend of the punctual estimation). (DOCX) [file pone.0098011.s006.docx]

**Table ST 4. Measures of performance for parameters estimation for the simulated example 2.** The two values displayed for coverage correspond to the coverage of 50% (left) and 95% (right) and not to the values corresponding to mode and median (coverage does not depend of the punctual estimation).

| ***Parameters*** *Statistics* | ***BaySICS*** | | ***BSSC + Rabc*** | ***DIYABC*** | |
| --- | --- | --- | --- | --- | --- |
|  | Mode | Median | Median | Mode | Median |
| ***N_e_*_1_** |  |  |  |  |  |
| *Relative bias* | -0.0573 | 0.2359 | 0.2031 | 0.0406 | 0.2262 |
| *RRMSE* | 0.5922 | 0.8733 | 0.6834 | 5.0680 | 5.6330 |
| *Coverage 50/95* | 0.5262 | 0.9644 | - | 0.4830 | 0.9080 |
| *Factor 2* | 0.7236 | 0.8280 | 0.8733 | 0.7350 | 0.7990 |
| ***t*_1_** |  |  |  |  |  |
| *Relative bias* | 0.0054 | 0.2921 | 0.1428 | 0.0044 | 0.2456 |
| *RRMSE* | 0.7426 | 1.1021 | 0.5743 | 8.9920 | 7.9810 |
| *Coverage 50/95* | 0.5169 | 0.9544 | - | 0.4640 | 0.9010 |
| *Factor 2* | 0.6984 | 0.8079 | 0.9010 | 0.7030 | 0.7840 |
| ***N_e_*_2_** |  |  |  |  |  |
| *Relative bias* | 0.0631 | 0.1846 | 0.2194 | -0.1592 | 0.1710 |
| *RRMSE* | 0.6317 | 0.7417 | 0.7230 | 18.2670 | 15.8700 |
| *Coverage 50/95* | 0.5390 | 0.9613 | - | 0.4690 | 0.8940 |
| *Factor 2* | 0.8196 | 0.8660 | 0.8813 | 0.6480 | 0.7710 |
| ***t*_2_** |  |  |  |  |  |
| *Relative bias* | 0.0483 | 0.0247 | 0.0374 | 0.0551 | 0.0231 |
| *RRMSE* | 0.2153 | 0.2600 | 0.1975 | 2.8300 | 3.1320 |
| *Coverage 50/95* | 0.5319 | 0.9520 | - | 0.4860 | 0.8900 |
| *Factor 2* | 0.9985 | 0.9976 | 1.0000 | 0.9800 | 0.9810 |
| ***N_e3_*** |  |  |  |  |  |
| *Relative bias* | -0.0069 | 0.0672 | 0.0598 | 0.0690 | 0.2287 |
| *RRMSE* | 0.3546 | 0.5679 | 0.3041 | 15.8580 | 13.7390 |
| *Coverage 50/95* | 0.5429 | 0.9688 | - | 0.4630 | 0.9140 |
| *Factor 2* | 0.9442 | 0.9530 | 0.9842 | 0.8720 | 0.8900 |
