# Supplementary material for: Back to BaySICS: A User-Friendly Program for Bayesian Statistical Inference from Coalescent Simulations
Source: PLoS One. 2014 May 27;9(5):e98011. doi: 10.1371/journal.pone.0098011 (PMC4035278; doi:10.1371/journal.pone.0098011)
Supplement: Table S5 — Measures of performance for parameters estimation for the simulated example 3. The two values displayed for coverage correspond to the coverage of 50% (left) and 95% (right) and not to the values corresponding to mode and median, (coverage does not depend of the punctual estimation). In both simulated example 2 and simulated example 3, the estimates obtained with DIYABC presented important inconsistencies when the number of iterations (PODs) varied: the ones obtained for 10 000 iterations were one order of magnitude larger than those obtained with 1 000 iterations. In addition, most of the values are much more similar between BSSC+Rabc and BaySICS. Those facts could be indicative of a numerical issue of the type of “catastrophic cancelation”. So the true performance measures for DIYABC clearly should be much better for those parameters. (DOCX) [file pone.0098011.s007.docx]

**Table ST 5. Measures of performance for parameters estimation for the simulated example 3.** The two values displayed for coverage correspond to the coverage of 50% (left) and 95% (right) and not to the values corresponding to mode and median, (coverage does not depend of the punctual estimation). In both simulated example 2 and simulated example 3, the estimates obtained with DIYABC presented important inconsistencies when the number of iterations (PODs) varied: the ones obtained for 10 000 iterations were one order of magnitude larger than those obtained with 1 000 iterations. In addition, most of the values are much more similar between BSSC+Rabc and BaySICS. Those facts could be indicative of a numerical issue of the type of “catastrophic cancelation”. So the true performance measures for DIYABC clearly should be much better for those parameters.

| ***Parameters***  *Statistics* | ***BaySICS*** | | ***BSSC + Rabc*** | ***DIYABC*** | |
| --- | --- | --- | --- | --- | --- |
|  | Mode | Median | Median | Mode | Median |
| ***N_e_*_1_** |  |  |  |  |  |
| *Relative bias* | 0.2073 | 0.3665 | 0.3034 | 3.6130 | 2.9935 |
| *RRMSE* | 1.0772 | 1.0273 | 0.9320 | 3.5750 | 2.9870 |
| *Coverage 50/95* | 0.5126 | 0.9580 | - | 0.4840 | 0.9040 |
| *Factor 2* | 0.6364 | 0.8154 | 0.8024 | 0.4920 | 0.7550 |
| ***N_e_*_2_** |  |  |  |  |  |
| *Relative bias* | 0.0619 | 0.2156 | 0.1721 | 3.0232 | 2.3799 |
| *RRMSE* | 0.6441 | 0.7125 | 0.6458 | 2.9990 | 2.3760 |
| *Coverage 50/95* | 0.5532 | 0.9620 | - | 0.4790 | 0.9090 |
| *Factor 2* | 0.7986 | 0.8656 | 0.8647 | 0.6950 | 0.7760 |
| ***N_e_*_3_** |  |  |  |  |  |
| *Relative bias* | 0.0656 | 0.0949 | 0.0777 | 1.7225 | 1.2901 |
| *RRMSE* | 0.4810 | 0.4797 | 0.3984 | 1.7100 | 1.2880 |
| *Coverage 50/95* | 0.5400 | 0.9608 | - | 0.4790 | 0.9170 |
| *Factor 2* | 0.9026 | 0.9270 | 0.9569 | 0.8820 | 0.8980 |
| ***t*_1_** |  |  |  |  |  |
| *Relative bias* | 0.0105 | 0.1543 | 0.0967 | 2.3319 | 1.7985 |
| *RRMSE* | 0.5548 | 0.6072 | 0.4517 | 2.3280 | 1.7980 |
| *Coverage 50/95* | 0.5572 | 0.9670 | - | 0.4830 | 0.9120 |
| *Factor 2* | 0.8448 | 0.8956 | 0.9322 | 0.8220 | 0.8450 |
| ***N_e_*_4_** |  |  |  |  |  |
| *Relative bias* | 0.4365 | 0.3858 | 0.4172 | 2.1633 | 1.7051 |
| *RRMSE* | 1.3533 | 1.0739 | 1.1130 | 2.1490 | 1.7030 |
| *Coverage 50/95* | 0.4994 | 0.9594 | - | 0.4820 | 0.9010 |
| *Factor 2* | 0.6558 | 0.8160 | 0.8049 | 0.6320 | 0.7530 |
| ***t*_2_** |  |  |  |  |  |
| *Relative bias* | 0.0181 | 0.0341 | 0.0401 | 2.8404 | 2.1258 |
| *RRMSE* | 0.2692 | 0.2062 | 0.2062 | 2.8180 | 2.1220 |
| *Coverage 50/95* | 0.5030 | 0.9572 | - | 0.4870 | 0.9130 |
| *Factor 2* | 1.0000 | 1.0000 | 1.0000 | 0.9630 | 0.9640 |
| ***N_e_*_5_** |  |  |  |  |  |
| *Relative bias* | -0.0442 | 0.1485 | 0.1816 | 3.1065 | 2.3947 |
| *RRMSE* | 0.4735 | 0.5839 | 0.6826 | 3.1000 | 2.3930 |
| *Coverage 50/95* | 0.5364 | 0.9560 | - | 0.4880 | 0.9050 |
| *Factor 2* | 0.8296 | 0.8746 | 0.8426 | 0.6700 | 0.7620 |
