# Supplementary material for: Back to BaySICS: A User-Friendly Program for Bayesian Statistical Inference from Coalescent Simulations
Source: PLoS One. 2014 May 27;9(5):e98011. doi: 10.1371/journal.pone.0098011 (PMC4035278; doi:10.1371/journal.pone.0098011)
Supplement: Box S1 — Input file for simulation of the Simulated Example 1 in BaySICS. (DOCX) [file pone.0098011.s008.docx]

**Box SB 1. Input file for simulation of the Simulated Example 1 in BaySICS.**

**1 1 1 51**

**51 1 0**

**Prior 0.0 1**

**Prior Prior 0.0 1 1 1.0 1.0 2**

**0**

**1.0 15**

**1000 0.875**

**0.15**

**0.1 0.1 0.6 0.2**

**A01 1 0**

**A02 1 0**

**A03 1 0**

**A04 1 0**

**A05 1 0**

**A06 1 0**

**A07 1 0**

**A08 1 0**

**A09 1 0**

**A10 1 0**

**A11 1 0**

**A12 1 0**

**A13 1 0**

**A14 1 0**

**A15 1 0**

**A16 1 0**

**A17 1 0**

**A18 1 0**

**A19 1 0**

**A20 1 0**

**A21 1 0**

**A22 1 0**

**A23 1 0**

**A24 1 0**

**A25 1 0**

**A26 1 0**

**A27 1 0**

**A28 1 0**

**A29 1 0**

**A30 1 0**

**A31 1 0**

**A32 1 0**

**A33 1 0**

**A34 1 0**

**A35 1 0**

**A36 1 0**

**A37 1 0**

**A38 1 0**

**A39 1 0**

**A40 1 0**

**A41 1 0**

**A42 1 0**

**A43 1 0**

**A44 1 0**

**A45 1 0**

**A46 1 0**

**A47 1 0**

**A48 1 0**

**A49 1 0**

**A50 1 0**

**A51 1 0**

**Uniform + 10000 100000**

**Uniform + 2500 25000**

**Uniform + 1000 10000**
