# Supplementary material for: Back to BaySICS: A User-Friendly Program for Bayesian Statistical Inference from Coalescent Simulations
Source: PLoS One. 2014 May 27;9(5):e98011. doi: 10.1371/journal.pone.0098011 (PMC4035278; doi:10.1371/journal.pone.0098011)
Supplement: Box S2 — Input file for simulation of the Simulated Example 2 in BaySICS. (DOCX) [file pone.0098011.s009.docx]

**Box SB 2. Input file for simulation of the Simulated Example 2 in BaySICS.**

**3 1 2 51**

**17 1 0**

**17 1 15000**

**17 1 35000**

**Prior 0.0 1**

**Prior Prior 0.0 1 1 1.0 1.0 2**

**Prior Prior 0.0 2 2 1.0 1.0 3**

**0**

**1.0 15**

**1000 0.875**

**0.15**

**0.1 0.1 0.6 0.2**

**A01 1 0**

**A02 1 0**

**A03 1 0**

**A04 1 0**

**A05 1 0**

**A06 1 0**

**A07 1 0**

**A08 1 0**

**A09 1 0**

**A10 1 0**

**A11 1 0**

**A12 1 0**

**A13 1 0**

**A14 1 0**

**A15 1 0**

**A16 1 0**

**A17 1 0**

**A18 2 0**

**A19 2 0**

**A20 2 0**

**A21 2 0**

**A22 2 0**

**A23 2 0**

**A24 2 0**

**A25 2 0**

**A26 2 0**

**A27 2 0**

**A28 2 0**

**A29 2 0**

**A30 2 0**

**A31 2 0**

**A32 2 0**

**A33 2 0**

**A34 2 0**

**A35 3 0**

**A36 3 0**

**A37 3 0**

**A38 3 0**

**A39 3 0**

**A40 3 0**

**A41 3 0**

**A42 3 0**

**A43 3 0**

**A44 3 0**

**A45 3 0**

**A46 3 0**

**A47 3 0**

**A48 3 0**

**A49 3 0**

**A50 3 0**

**A51 3 0**

Box SB 2. (Continuation).

**Uniform + 5000 50000**

**Uniform + 1000 10000**

**Uniform + 500 5000**

**Uniform + 15001 30000**

**Uniform + 5000 50000**
