# Supplementary material for: Back to BaySICS: A User-Friendly Program for Bayesian Statistical Inference from Coalescent Simulations
Source: PLoS One. 2014 May 27;9(5):e98011. doi: 10.1371/journal.pone.0098011 (PMC4035278; doi:10.1371/journal.pone.0098011)
Supplement: Box S3 — Input file for simulation of the Simulated Example 3 in BaySICS. (DOCX) [file pone.0098011.s010.docx]

**Box SB 3. Input file for simulation of the Simulated Example 3 in BaySICS.**

**3 3 2 51**

**17 1 0**

**17 2 2500**

**17 3 5000**

**Prior 0.00 1**

**Prior 0.00 2**

**Prior 0.00 3**

**Prior Prior 0.0 1 2 1.0 1.0 4**

**Prior Prior 0.0 4 3 1.0 1.0 5**

**0**

**1.0 15**

**1000 0.875**

**0.15**

**0.1 0.1 0.6 0.2**

**A01 1 0**

**A02 1 0**

**A03 1 0**

**A04 1 0**

**A05 1 0**

**A06 1 0**

**A07 1 0**

**A08 1 0**

**A09 1 0**

**A10 1 0**

**A11 1 0**

**A12 1 0**

**A13 1 0**

**A14 1 0**

**A15 1 0**

**A16 1 0**

**A17 1 0**

**B01 2 0**

**B02 2 0**

**B03 2 0**

**B04 2 0**

**B05 2 0**

**B06 2 0**

**B07 2 0**

**B08 2 0**

**B09 2 0**

**B10 2 0**

**B11 2 0**

**B12 2 0**

**B13 2 0**

**B14 2 0**

**B15 2 0**

**B16 2 0**

**B17 2 0**

**C01 3 0**

**C02 3 0**

**C03 3 0**

**C04 3 0**

**C05 3 0**

**C06 3 0**

**C07 3 0**

**C08 3 0**

**C09 3 0**

**C10 3 0**

**C11 3 0**

**C12 3 0**

**C13 3 0**

**C14 3 0**

Box SB 3. (Continuation).

**C15 3 0**

**C16 3 0**

**C17 3 0**

**Uniform + 100 1000**

**Uniform + 1000 10000**

**Uniform + 10000 100000**

**Uniform + 2501 25000**

**Uniform + 100 1000**

**Uniform + 25000 50000**

**Uniform + 10000 100000**
