# Supplementary material for: Back to BaySICS: A User-Friendly Program for Bayesian Statistical Inference from Coalescent Simulations
Source: PLoS One. 2014 May 27;9(5):e98011. doi: 10.1371/journal.pone.0098011 (PMC4035278; doi:10.1371/journal.pone.0098011)
Supplement: Box S4 — Input file for simulation of the Simulated Example 1 in BSSC. (DOCX) [file pone.0098011.s011.docx]

**Box SB 4. Input file for simulation of the Simulated Example 1 in BSSC.**

**//Simulated Example 1**

**1 population**

**//Deme sizes (haploid number of genes)**

**{U:10000,100000}**

**//Sample sizes, ages, deme, stat_grp**

**1 sample groups**

**51 0 0 0**

**//Growth rates**

**0**

**//Number of migration matrices : If 0 : No migration between demes**

**0**

**//Historical event: time, source, sink, migrants, new deme size, new growth rate, new migration matrix**

**1 events**

**{U:2500,25000} 0 0 1.0 {[4]/[1]} 0 0**

**//Mutation rate per generation for the whole sequence**

**0.00015**

**//Number of loci**

**1000**

**//Data type : either DNA, RFLP, or MICROSAT : If DNA, second term is the //transition bias**

**DNA 0.875**

**//Mutation rates gamma distribution shape parameter**

**0.15 0**

**{U:1000,10000}**
