# Supplementary material for: Back to BaySICS: A User-Friendly Program for Bayesian Statistical Inference from Coalescent Simulations
Source: PLoS One. 2014 May 27;9(5):e98011. doi: 10.1371/journal.pone.0098011 (PMC4035278; doi:10.1371/journal.pone.0098011)
Supplement: Box S7 — Header file for simulation of the Simulated Example 1 in DIYABC. (DOCX) [file pone.0098011.s014.docx]

**Box SB 7. Header file for simulation of the Simulated Example 1 in DIYABC.**

**DIYABCSc1.txt**

**4 parameters and 5 summary statistics**

**1 scenarios: 3**

**scenario 1 [1.0] (3)**

**N1**

**0 sample 1**

**t varNe 1 N2**

**historical parameters priors (3,0)**

**N1 N UN[10000,100000,0.0,0.0]**

**t T UN[2500,25000,0.0,0.0]**

**N2 N UN[1000,10000,0.0,0.0]**

**loci description (1)**

**locus_S_M_1_ <M> [S] G1 1000**

**group priors (1)**

**group G1 [S]**

**MEANMU UN[0.00000015,0.00000015,5E-9,2]**

**GAMMU GA[1.00E-9,1.00E-6,Mean_u,0.15]**

**MEANK1 UN[0.050,20,10,2]**

**GAMK1 GA[0.050,20,Mean_k1,0]**

**MEANK2 UN[0.050,20,10,2]**

**GAMK2 GA[0.050,20,Mean_k2,2]**

**MODEL K2P 0 0.15**

**group summary statistics (5)**

**group G1 [S] (5)**

**NHA 1**

**NSS 1**

**MPD 1**

**VPD 1**

**DTA 1**

**scenario N1 t N2 k1seq_1 NHA_1_1 NSS_1_1 MPD_1_1 VPD_1_1 DTA_1_1**
