# Supplementary material for: Back to BaySICS: A User-Friendly Program for Bayesian Statistical Inference from Coalescent Simulations
Source: PLoS One. 2014 May 27;9(5):e98011. doi: 10.1371/journal.pone.0098011 (PMC4035278; doi:10.1371/journal.pone.0098011)
Supplement: Box S8 — Header file for simulation of the Simulated Example 2 in DIYABC. (DOCX) [file pone.0098011.s015.docx]

**Box SB 8. Header file for simulation of the Simulated Example 2 in DIYABC.**

**DIYABCSc2.txt**

**6 parameters and 19 summary statistics**

**1 scenarios: 6**

**scenario 1 [1.0] (5)**

**N1**

**0 sample 1**

**15000 sample 1**

**35000 sample 1**

**t1 varNe 1 N2**

**t2 varNe 1 N3**

**historical parameters priors (5,0)**

**N1 N UN[5000,50000,0.0,0.0]**

**t1 T UN[1000,10000,0.0,0.0]**

**N2 N UN[500,5000,0.0,0.0]**

**t2 T UN[15001,30000,0.0,0.0]**

**N3 N UN[5000,50000,0.0,0.0]**

**loci description (1)**

**locus_S_M_1_ <M> [S] G1 1000**

**group priors (1)**

**group G1 [S]**

**MEANMU UN[0.00000015,0.00000015,5E-9,2]**

**GAMMU GA[1.00E-9,1.00E-6,Mean_u,0.15]**

**MEANK1 UN[0.050,20,10,2]**

**GAMK1 GA[0.050,20,Mean_k1,0]**

**MEANK2 UN[0.050,20,10,2]**

**GAMK2 GA[0.050,20,Mean_k2,2]**

**MODEL K2P 0 0.15**

**group summary statistics (19)**

**group G1 [S] (19)**

**NHA 1 2 3**

**NSS 1 2 3**

**MPD 1 2 3**

**DTA 1 2 3**

**PSS 1 2 3**

**MP2 1&2 2&3**

**HST 1&2 2&3**

**scenario N1 t1 N2 t2 N3 k1seq_1 NHA_1_1 NHA_1_2 NHA_1_3 NSS_1_1**

**NSS_1_2 NSS_1_3 MPD_1_1 MPD_1_2 MPD_1_3 DTA_1_1 DTA_1_2 DTA_1_3**

**PSS_1_1 PSS_1_2 PSS_1_3 MP2_1_1&2 MP2_1_2&3 HST_1_1&2 HST_1_2&3**
